# Selenomethionine Ameliorates Neuropathology in the Olfactory Bulb of a Triple Transgenic Mouse Model of Alzheimer’s Disease

**DOI:** 10.3390/ijms17101595

**Published:** 2016-09-27

**Authors:** Zhong-Hao Zhang, Chen Chen, Qiu-Yan Wu, Rui Zheng, Yao Chen, Qiong Liu, Jia-Zuan Ni, Guo-Li Song

**Affiliations:** 1 Changchun Institute of Applied Chemistry, University of Chinese Academy of Sciences, Changchun 130022, China; zhangzhonghao@email.szu.edu.cn (Z.-H.Z.); jzni@szu.edu.cn (J.-Z.N.); 2 Shenzhen Key Laboratory of Marine Bioresources and Ecology, College of Life Sciences and Oceanography, Shenzhen University, Shenzhen 518060, China; chenchen198951@163.com (C.C.); happy_wuqiuyan@126.com (Q.-Y.W.); zhengrui_210@163.com (R.Z.); yaochen913@163.com (Y.C.); liuqiong@szu.edu.cn (Q.L.)

**Keywords:** olfactory dysfunction, Alzheimer’s disease, CDK5, tau, selenomethionine

## Abstract

Olfactory dysfunction is an early and common symptom in Alzheimer′s disease (AD) and is reported to be related to several pathologic changes, including the deposition of Aβ and hyperphosphorylated tau protein as well as synaptic impairment. Selenomethionine (Se-Met), the major form of selenium in animals and humans, may be a promising therapeutic option for AD as it decreases the deposition of Aβ and tau hyperphosphorylation in a triple transgenic mouse model of AD (3× Tg-AD). In this study, 4-month-old AD mice were treated with 6 µg/mL Se-Met in drinking water for 12 weeks and the effect of Se-Met on neuropathological deficits in olfactory bulb (OB) of 3× Tg-AD mice was investigated. The administration of Se-Met effectively decreased the production and deposition of Aβ by inhibiting β-site amyloid precursor protein cleaving enzyme 1 (BACE1)-regulated amyloid precursor protein (APP) processing and reduced the level of total tau and phosphorylated tau, which depended on depressing the activity and expression of glycogen synthase kinase-3*β* (GSK-3*β*) and cyclin-dependent kinase 5 (CDK5). Meanwhile, Se-Met reduced glial activation, relieved neuroinflammation and attenuated neuronal cell death in the OB of AD mice. So Se-Met could improve pathologic changes of AD in the OB, which further demonstrated the potential therapeutic effect of Se-Met in AD.

## 1. Introduction

Alzheimer′s disease (AD), which is pathologically defined by the presence of amyloid-β senile plaques (SPs), neurofibrillary tangles (NFTs), and neuronal loss within the brain, is an age-related neurodegenerative disease wherein patients suffer from sensory, motor, and cognitive loss [1,2]. Among human sensory systems, the olfactory system is a unique brain system that is directly connected to the brain [3]. Previous studies have demonstrated that olfactory function decreases with aging and the decline in olfaction is part of the clinical phenotype in neurodegenerative disorders, including AD. The functions of odor detection, discrimination, and identification were affected earlier than the decrease in cognitive ability [4]. In AD mice, olfactory deficits also occurred before the clinical onset of cognitive deficits and coincided with AD pathology [5,6,7]. Olfactory dysfunction is prevalent in patients with AD and mild cognitive impairment [8], as the neuropathological abnormalities in both central and peripheral olfactory systems have been described in AD patients. Thus, olfactory dysfunction has been proposed as a potential biomarker for AD diagnosis [9,10].

The olfactory bulb (OB), as a cortical sensory structure, is the first synaptic relay station of olfactory perception in the brain, directly receiving inputs from the olfactory sensory neurons located in the nasal cavity [11]. Olfaction involves distinct processes spanning from sensory neuron input to the OB, which involves decoding and plasticity in the prepiriform cortex, and ultimately downstream neurons in the hippocampus [12]. Neuropathological studies have found that the potential origins of olfactory dysfunction in AD might be attributable to the presence of NFTs, senile Aβ plaques in the OB, the anterior olfactory nucleus, the prepiriform cortex, the entorhinal cortex, the amygdala and the hippocampus [13,14]. Moreover, the presence and severity of β-amyloid and hyperphosphorylated tau in the OB can reflect the severity of AD pathologies in other brain regions [15]. Together, Aβ and tau pathology in OB can serve as a crucial pathogenic and diagnostic hallmark of olfactory dysfunction and AD. Detecting the pathological changes of AD in the OB after pharmacological intervention might be a better assessment compared with assessments using other brain regions during the early AD stage or after short-term treatment.

Selenium (Se) is a vital non-metallic trace element that is rich in the brain and possesses beneficial biochemical and pharmacological properties (including antioxidation, anticancer, and anti-inflammatory) [16]. It has been shown in our previous study that selenomethionine (Se-Met), a major form of Se in organisms, effectively increase oxidation resistance as well as reduce the production and deposition of Aβ and tau hyperphosphorylation in the cortex and hippocampus of a triple transgenic mouse model of AD (3× Tg-AD), thereby suggesting a promising therapeutic option for AD [17]. In this study, we further explore the effect of Se-Met treatment on neuropathological changes in the OB of 3× Tg-AD mice at 4 months of age. In the meantime, we also attempted to demonstrate a feasible way that AD pathology in the OB is also sensitive to pharmacological intervention beyond the cortex and hippocampus.

## 2. Results

### 2.1. Se-Met Decreased the Production and Deposition of Aβ by Inhibiting Level of APP and BACE1-Mediated APP Processing

**Figure 1:**
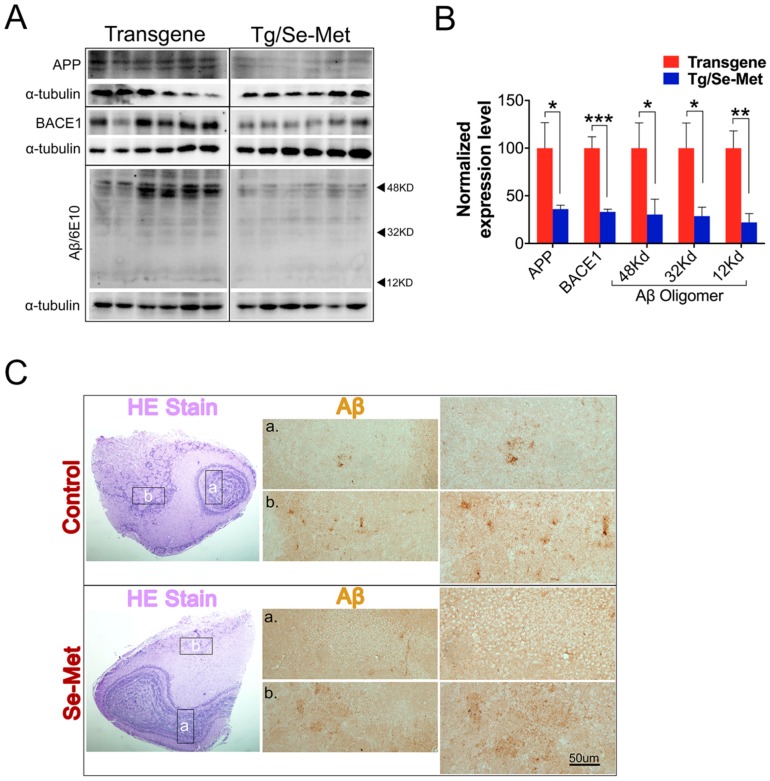
Treatment with Se-Met regulated APP processing by reducing the levels of β-site amyloid precursor protein cleaving enzyme (BACE1) and APP so as to decrease the burden of amyloid production and deposition of Aβ in the OB of 3× Tg-AD mice. (**A**) The level of full-length APP (fAPP), BACE1, and Aβ in the OB were detected by western blot; (**B**) Quantitative analysis showed that Se-Met significantly reduced the levels of fAPP, BACE1, and Aβ oligomers (~12, ~32, and ~48 kD) in the OB of 3× Tg-AD mice. Quantitative results were normalized against the levels of α-tubulin. Values were expressed as percentages in comparison to the control (set to 100%) and presented as the group mean ± SEM (*n* = 6). * *p* < 0.05, ** *p* < 0.01 *** *p* < 0.001 vs. the control group; (**C**) Hematoxylin-eosin (HE) staining and immunohistochemical staining using antibody 6E10 revealed that Se-Met had noticeably reduced deposition of Aβ in all layers of the olfactory bulb (OB) compared with the control mice (a: high magnification images of Gl and Epl layers of OBs; b: high magnification images of MT and Ipl layers of OBs). Scale bars, 50 µm.

Characterization of Aβ burden is a valuable way to assess the pathological changes in the OB. To determine the effect of Se-Met on the production of Aβ, the expression of Aβ oligomers was detected by western blot using the specific antibody 6E10. Se-Met significantly reduced the production of Aβ oligomers (~12, 32 and 48 kD) in the OB tissue (12 kD: *p <* 0.05; 32 kD: *p <* 0.05; 48 kD: *p <* 0.01; Figure 1A,B). To further ascertain the result, the structure of OB was observed using hematoxylin-eosin (HE) staining and the expression of Aβ_1–42_ in OB was detected by immunohistochemistry. As shown in Figure 1C, the region in the b-pane is the glomerular (G) layer, which is the first receptor in odor information processing. There are four layers beneath the G layer, as shown in the a-pane, including the external plexiform (EP) layer, the mitral and tufted (MT) cell layer, and the inner plexiform (IP) layer. The center of the OB is the granule cell (Gr) layer, and all layers of the OB are essential in odor information processing [1]. Se-Met-treated mice had obviously less deposition of Aβ in all layers of OB compared with the control mice. These results demonstrated that Se-Met could significantly decrease the deposition of Aβ.

**Figure 2:**
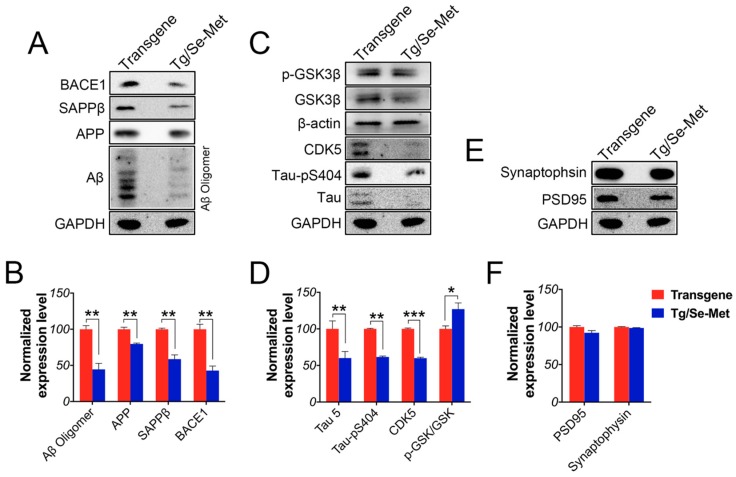
Se-Met treatment improved AD related pathology in the primary neurons of the OB. (**A**–**F**) Western blot analysis of AD-related pathological proteins in the primary neurons of the OB indicated that Se-Met could significantly decrease the levels of Aβ oligomers, APP, SAPPβ, BACE1 and total tau protein, inhibited hyperphosphorylation of tau at Ser404, and reduce the level of CDK5 and the activity of GSK3*β*, without increasing the expression levels of synaptic proteins. Quantitative results were normalized against the levels of *β*-actin or GAPDH. Values are expressed as percentages in comparison with the control (set to 100%) and presented as the group mean ± SEM (*n* = 3). * *p* < 0.05, ** *p* < 0.01, *** *p* < 0.001 vs. the transgene group.

To explore the mechanism of reduced deposition of Aβ by Se-Met, the amyloidogenic APP processing pathway was investigated. First, the expression level of full-length APP (fAPP) was assessed using western blot. There was a significant decrease in the expression level of fAPP in the OB after treatment with Se-Met (*p <* 0.05; Figure 1A,B). In the amyloidogenic pathway, BACE1 is responsible for the initiation of Aβ generation. BACE1 cuts APP to generate the N-terminus of Aβ by producing a membrane-bound C-terminal fragment named C99 (β-CTF) before γ-secretase cleaves C99 to release the mature Aβ peptide. Thus, we detected the expression of BACE1. In doing so, the results showed that Se-Met significantly inhibited BACE1 expression in the OB of 3× Tg-AD mice (*p <* 0.05; Figure 1A,B). Meanwhile, in Se-Met-treated primary OB neurons, besides decreased expression of APP and BACE-1, the level of sAPPβ also markedly decreased compared with the control group (Figure 2A). Together, it suggests that Se-Met reduces the deposition and generation of Aβ by down-regulating the expression of fAPP and BACE1.

### 2.2. Se-Met Reduced the Level of Total Tau and Phosphorylation of Tau at Ser404, Ser422 in the OB

**Figure 3:**
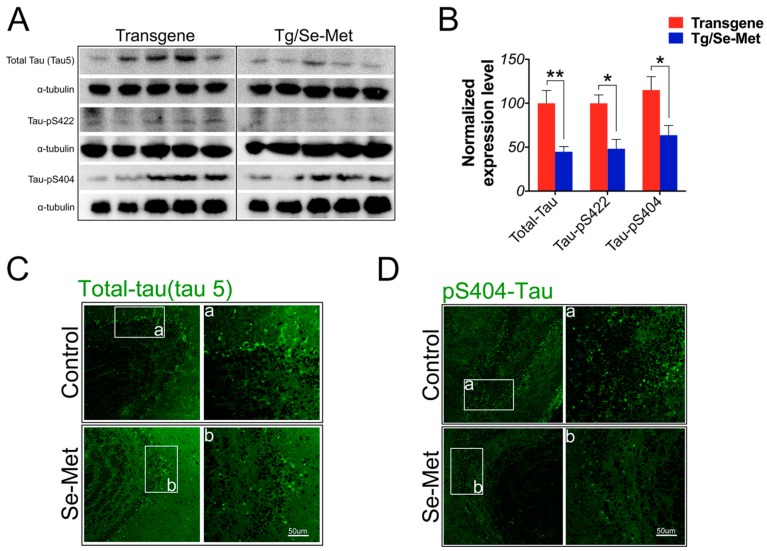
Se-Met treatment attenuated tau expression and hyperphosphorylation in 3× Tg-AD mice. (**A**,**B**) Western blot analysis of total tau and phosphorylated tau at Ser404 and Ser422 showed that the levels of total tau, tau-pS404, and tau-pS422 decreased significantly in the OB of Se-Met-treated mice. Quantitative results were normalized against the levels of α-tubulin. Values were expressed as percentages in comparison with the control (set to 100%) and presented as the group mean ± SEM (*n* = 6). * *p* < 0.05, ** *p* < 0.01 vs. the control group; (**C**,**D**) Immunofluorescence results showed Se-Met could reduce the pathological epitope of total tau and tau-pS404 compared with the control mice in the OB (a,b: high magnification images of the regions of OB). Scale bar, 50 µm.

Hyperphosphorylation of tau results in an impairment in the function of tau and in the formation of NFTs in AD [18]. Thus, it plays an important role in the pathogenesis of Alzheimer′s disease. In the present study, the levels of total tau and phosphorylation of tau were examined by western blot using antibodies directed against total tau protein and different phosphorylation sites on tau including Ser404 and 422. As shown in Figure 3A,B, a significant decrease in the level of total tau and phosphorylation of tau at Ser404 and 422 sites were observed in the OB of Se-Met-treated mice compare to that of control mice (total tau: *p <* 0.01; tau-pS404: *p <* 0.05; tau-pS422: *p <* 0.05; Figure 3A,B). Meanwhile, further detection using immunofluorescence staining also showed that the immunoreactive region of tau and phosphorylated tau at Ser404 noticeably decreased in Se-Met-treated mice compared with the control group (Figure 3C,D). Thus, Se-Met could decrease both the level of total tau and phosphorylation of tau in the OB.

### 2.3. Effect of Se-Met on the Activity of CDK5, GSK-3β, PP2A, and Akt in the OB

**Figure 4:**
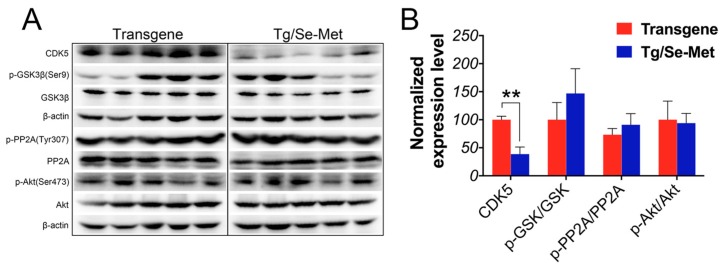
Se-Met treatment reduced the level of CDK5, inhibited the activity of GSK-3*β*. (**A**) The level of CDK5, GSK-3*β*, phosphorylated GSK-3*β* (Ser9), PP2A, phosphorylated PP2A (Tyr307), Akt, and phosphorylated Akt (Ser473) in the OB were detected using western blot; (**B**) Quantitative analysis indicated that the level of CDK-5 significantly decreased in Se-Met-treated mice and that Se-Met could also increase the relative ratio of pGSK-3*β*/GSK-3*β* with no significant difference. Quantitative results were normalized against the levels of *β*-actin. Values were expressed as percentages in comparison with the control (set to 100%) and presented as the group mean ± SEM (*n* = 5). ** *p* < 0.01 vs. the control group.

To further elucidate the mechanism of Se-Met in inhibiting Aβ production and tau hyperphosphorylation in the OB of 3× Tg-AD mice, we assessed the expressions of GSK-3*β*, phospho-GSK-3*β* (pGSK-3*β*), PP2A, phospho-PP2A (pPP2A), Akt, phospho-Akt (pAkt), and CDK5. As shown in Figure 4A,B, the level of CDK-5 significantly decreased in Se-Met-treated mice (*p <* 0.01). It was found that there was a negative correlation between the activity of GSK-3*β* and the ratio of pGSK-3*β*/GSK-3*β*. Se-Met treatment could increase the relative ratio of pGSK-3*β*/GSK-3*β*, although the difference was not statistically significant due to inter-individual variability. Nevertheless, there were no obvious changes in the ratios of pPP2A/PP2A and pAkt/Akt compared with the control mice.

### 2.4. Se-Met Treatment Inhibited the Activation of Glials in OB

**Figure 5:**
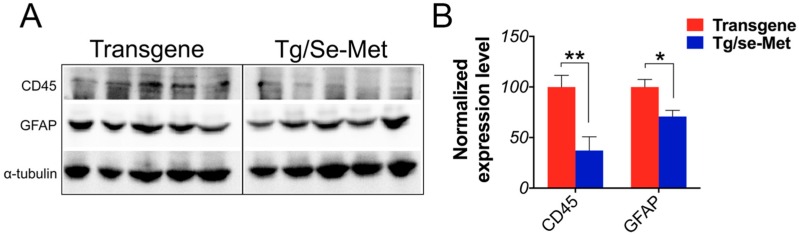
Se-Met treatment reduced glial activation in the OB of 3× Tg-AD mice. (**A**,**B**) Western blot analysis of GFAP and CD45 showed that Se-Met could significantly decrease the expression level of glial fibrillary acidic protein (GFAP) and CD45 in the OB. Quantitative results were normalized against the levels of α-tubulin. Values were expressed as percentages in comparison with the control (set to 100%) and presented as the group mean ± SEM (*n* = 5). * *p* < 0.05, ** *p* < 0.01 vs. the control group.

Gliocytes were known as the first line of defense and the main effectors in the inflammatory process of the central nervous system. Activated astrocytes and microglia were observed to be abundant and closely associated with Aβ deposits in AD mice. To investigate whether Se-Met treatment has anti-inflammatory effects in the OB of 3× Tg-AD mice, we detected the levels of astrocyte and microglia reactivity by western blot with an anti-GFAP antibody for astrocytes and an anti-CD45 antibody for microglia. It has been shown that Se-Met significantly reduced both GFAP and CD45 immunoreactivity in the OB of 3× Tg-AD mice compared with the control mice (CD45: *p <* 0.01; GFAP: *p <* 0.05; Figure 5A,B).

### 2.5. Se-Met Attenuated Neuronal Cell Death in the OB of 3× Tg-AD Mice

**Figure 6:**
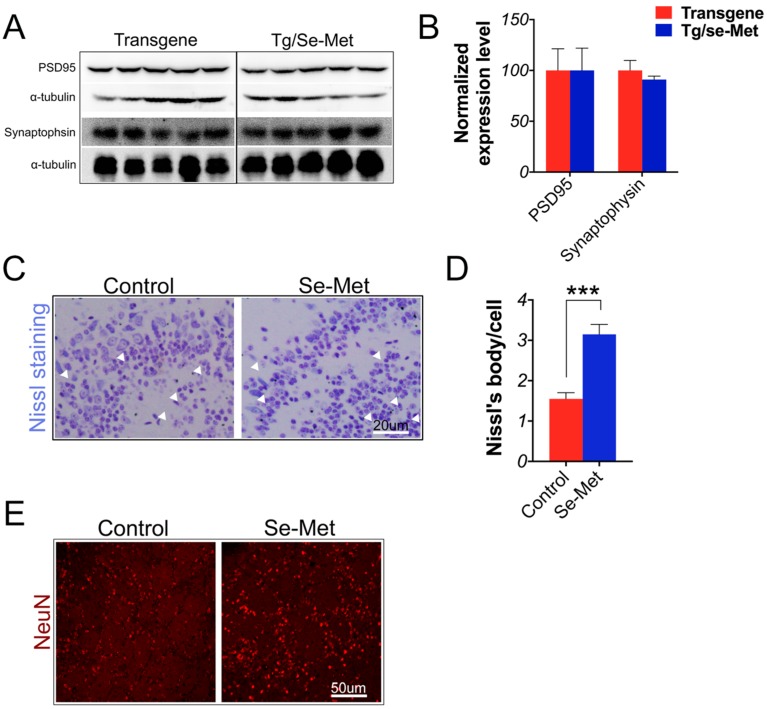
Se-Met treatment reduced neuronal cell death without increasing the level of synaptic proteins in the OB of 3× Tg-AD mice. (**A**,**B**) Western blot analysis of PSD95 and synaptophysin showed that there were no significant differences in the level of these two proteins between Se-Met-treated and the control mice in the OB. Quantitative results were normalized against the levels of *α*-tubulin. Values were expressed as percentages in comparison with the control (set to 100%) and presented as the group mean ± SEM (*n* = 5); (**C**–**E**) Nissl staining and immunofluorescence staining using specific antibody of NeuN were applied to analyze the density and activity of neurons (as indicated by the white arrows) in the OB of 3× Tg-AD mice. Quantitative results showed Se-Met treatment significantly increase the density of Nissl bodies. Values represent group mean ± SEM (*n* = 3), *** *p* < 0.001 vs. the control group. Scale bar, 50 µm.

Synaptic deficit (i.e., reduction of synaptic proteins and abnormal synaptic plasticity processes) was another primary characteristic of AD [19]. Thus, to determine whether Se-Met treatment could rescue the synaptic deficit in OB, the expression of two synaptic proteins (synaptophysin and post-synaptic density protein 95 (PSD95)) were measured using western blot. The results showed that there were no significant differences in the level of these two proteins between Se-Met-treated and the control mice (Figure 6A,B). The death of neurons could directly result in a deficit in learning and memory in AD. Therefore, Nissl staining and immunofluorescence staining using specific antibodies against NeuN were applied to analyze the density and activity of neurons in the OB of AD mice. As shown in Figure 6C,D, neurons displayed healthy morphologies with less degeneration or pyknosis and had more abundant Nissl bodies in Se-Met-treated mice compared with the control mice (*p <* 0.001). Moreover, Se-Met treatment noticeably increased the density of neurons in the OB, as indicated by immunofluorescence staining with anti-NeuN antibody (Figure 6E).

### 2.6. Se-Met Improved AD Related Pathology in the Primary Neuron of OB

Finally, to further verify effects of Se-Met on the pathology of AD, primary neurons from the OB were isolated and treated with Se-Met. The results were similar to those found in vivo: Se-Met significantly reduced the levels of Aβ oligomers, APP, sAPPβ, BACE1 and total tau protein, inhibited hyperphosphorylation of tau at Ser404, as well as decreased the activity of GSK-3β and CDK5. Meanwhile, there were also no significant differences in the levels of synaptic proteins between Se-Met-treated and the control neurons (Aβ oligomers, APP, sAPPβ, BACE1, tau 5, tau-pS404: *p* < 0.01; p-GSK/GSK: *p* < 0.05; CDK5: *p* < 0.001; Figure 2A–F).

## 3. Discussion

According to the current hypothesis, Aβ and tau have essential roles in the pathogenesis of AD, which is characterized by the accumulation of extracellular SPs and intracellular NFTs [20]. Previously, we showed that long-term Se-Met treatment decreased the production and deposition of Aβ and hyperphosphorylation of tau in the cortex and hippocampus, and improved memory deficits in 3× Tg-AD mice. This demonstrated the therapeutic potential of Se-Met in AD [17]. However, olfactory memory deficits have also been found in these 3× Tg-AD mice [21]. Studies have shown that olfactory deficit is a major part of cognitive impairment in AD and is highly correlated with Aβ deposition and tau hyperphosphorylation [11], especially those occurring in the OBs beyond the cortex and hippocampus [22,23]. The OB is the first site for the processing of olfactory information in the brain and its deregulation is associated with AD. Furthermore, the dysfunction of neurons, including all regions and neuron types in the OB, is an early event in the pathogenesis of AD [24]. Therefore, in this study, we sought to clarify the effect of Se-Met on pathological changes in the OB.

It has been suggested that the potential origins of olfactory dysfunction may be the deposition and accumulation of Aβ and tau protein in the OB, olfactory tract, and olfactory cortex [25,26]. Studies have shown that Aβ deposition appeared in OB at 3–4 months of age and correlated with olfactory deficits in AD mice [27]. Cognitive deficits and synaptic loss correlate with soluble Aβ aggregates rather than SPs [28]. Aβ oligomers have a major role in the neurotoxic pathology of AD. A strong correlation also has been suggested between the accumulation of soluble Aβ in the OB and very early olfactory (including OB activity) dysfunction in both AD patients and transgenic mice [1,29]. Conversely, a reduced level of Aβ ameliorated the olfactory dysfunction observed in AD transgenic mice [27]. Therefore, as confirmed by the current results, Se-Met treatment reduced the expression of Aβ oligomers and Aβ deposition in the Gl, Epl, Ipl, MT, and Grl layers of the OB in 3× Tg-AD mice, which was exerted through the inhibition of the production of Aβ by downregulating the level of APP and BACE1. Meanwhile, although more NFT than plaques appeared in the olfactory system in AD patients, there have been a limited number of studies about the role of tau in OB. This may be due to previous reports that there is no tau immunoreactivity in OB of 3× Tg-AD mice [21]. Nevertheless, in this research, we detected the expression of both tau and phosphorylated tau in the OB although the expression levels are relatively lower than those in hippocampus and cortex. Furthermore, Se-Met treatment significantly reduced the level of tau and tau phosphorylated at Ser404 and 422 in the OB.

It is widely known that GSK-3β, a constitutively active proline-directed serine/threonine kinase, is the chief kinase that enhances the phosphorylation of tau and regulates the amyloidogenic process of APP through the modulation of BACE expression and γ-secretase function both in vitro and in vivo [30,31,32]. Furthermore, our previous studies have demonstrated that Se-Met reduced the level of tau phosphorylation by inhibiting the activity of GSK-3β in the cortex and hippocampus of 3× Tg-AD mice [17]. In the OB, Se-Met could also decrease the activity of GSK-3β (pGSK-3β/GSK-3β), but the difference did not reach a statistically significant level. Moreover, as the most important dephosphorylation enzyme, there were no changes in the level and activity of PP2A in the OB after the treatment of Se-Met as well as those of Akt, which is a crucial upstream kinase in Aβ and tau pathological pathway. Our findings regarding these enzymes in the OB were different from those in the cortex and hippocampus, which indicates that Se-Met might ameliorate Aβ and tau pathology through a different mechanism in the OB of AD mice.

Therefore, we continued to explore the expression of cyclin-dependent kinase 5 (Cdk5), another proline directed serine-threonine protein kinase, which has been demonstrated to play an important role in the physiological development of the central nervous system (such as neuronal migration, cytoskeleton remodeling, cortical cytoarchitecture, and synaptic plasticity), as well as the phosphorylation of many physiological relevant substrates [33,34]. The overexpression and activation of CDK5 induced an increase in NFTs and Aβ plaque in AD patients [35]. The activation of the calcium-dependent proteases in AD can finally induce a prolonged activation of CDK5 [36]. This overactivation of CDK5 resulted in the hyperphosphorylation of tau protein at different sites (such as Ser199 and 202, Thr205, 212, and 231, Ser235, 396, and 404), thereby resulting in PHF (paired helical filaments) formation and the subsequent formation of NFTs [37,38]. CDK5 knockdown reversed Aβ aggregation in the hippocampus by inhibiting the phosphorylation of GSK-3β at Ser9 and activating PP2A [39,40]. Interestingly, Se-Met could significantly decrease the expression of CDK5 in the OB. This might illuminate the mechanism by which Se-Met reduced the level of Aβ and hyperphosphorylation of tau protein, which was also proven by the in vivo experiment in primary OB neurons.

As the occurrence of cognitive impairment in Alzheimer′s disease ultimately depends on the loss of functional synapses or the death of neuron, rather than on Aβ plaques and NFTs themselves, we quantified the expression level of pre- and post-synaptic proteins in the OB of 3× Tg-AD mice to assess the efficacy of Se-Met [41]. The level of synaptophysin and PSD95 proteins were reduced in the hippocampus and cortex of AD brains [42,43]. However, there were no significant changes in the level of synaptophysin and PSD95 proteins in the OB after the treatment of Se-Met. It seems not to be consistent with our previous results that Se-Met could increase the expression level of synaptic proteins in the hippocampus and cortex. OB is severely damaged in the early stages of AD [9], and the cell density in the OB (particularly in the mitral cell layer) was much lower than that in WT (wild-type). As Se-Met significantly enhanced the activity and decreased the death of neuron cells, this might contribute to the neuroprotective effect of Se-Met.

It is known that oxidative damage is another crucial hallmark of AD beyond Aβ and tau pathology [44]. Neuroinflammation induced by excessive oxidative stress has been thought to play a pivotal role in brain host defense involved in neuronal loss of AD [45]. Chronic inflammation is characterized by longstanding microglia and astrocyte activation, which is followed by the sustained release of inflammatory mediators. Furthermore, activated astrocytes and microglia are found around Aβ and NFTs in AD [46,47]. The level of GFAP-positive astrocytes and CD45-positive microglia might indicate the severity of local inflammation response, which is correlated with memory impairment and neuronal loss. Se-Met was able to significantly decrease the expression levels of GFAP and CD45, which implied that it could inhibit the activated astrocytes and microglia and then restrain neuroinflammation in OB.

## 4. Materials and Methods

### 4.1. Animals and Treatment

3× Tg-AD mice (4-month-old), purchased from the Jackson Laboratory (Bar Harbor, Maine, ME, USA), were kept with accessible food and water under a 12-h light/dark cycle [48,49]. All animal experiments and procedures were approved by the Ethics Committee of Shenzhen University (Permit Number: AEWC-20140615-002). Mice (*n* = 24; 12 males and 12 females) were respectively treated with 6 µg/mL Se-Met (Sigma-Aldrich, Santa Clara, CA, USA) in drinking water or normal drinking water for 12 weeks and the body weight of each mouse was recorded every two weeks.

### 4.2. Immunofluorescent Staining and Histological Analysis

Mice were euthanized with anhydrous ether inhalation after treatment with Se-Met. Then, their OBs were rapidly removed and the left OBs were paraffin-embedded, and cut into 5-µm-thick sections and then mounted on glass slides. They were pretreated with 0.01 mol/L citrate buffer (pH = 6.0) in hyperthermy for 5 min and then blocked with 5% goat serum in phosphate buffer saline (PBS) for 10 min. These sections were further incubated with primary antibodies overnight at 4 °C, which was followed by incubation with secondary antibodies (1:500 in PBS) for 1 h at 37 °C. The primary antibodies used in this study are summarized in Table 1. The primary antibodies were detected using secondary antibodies (goat anti-mouse, goat anti-rabbit and goat anti-chicken; AlexaFluor-488 and -695, Multi Sciences Biotech, Beijing, China). Three equidistant sections were evaluated per animal. For analysis and quantification of immunoreactive areas, these sections were imaged with confocal microscopy (Olympus, Tokyo, Japan).

To assess the histopathological change and the neuronal cell loss, the slides were also subjected to Nissl staining. Briefly, 5-µm-thick sections were stained with a 0.5% cresyl violet solution for 10–15 min after washing twice for 15 min each in 0.01 M PBS and then dehydrated in graded ethanol, placed in Xylene and mounted on coverslips using the mounting medium. Quantification was carried out by counting the survival neurons which were characterized by the blue staining Nissl bodies under a microscope (Olympus).

### 4.3. Immunoblot Analysis

The OBs were homogenized in RIPA lysis buffer (Beyotime, Nanjing, China) with a protease inhibitor cocktail and phosphatase inhibitors (Roche, Basel, Switzerland). The samples were centrifuged at 13,000× *g* for 1.5 h at 4 °C and the supernatants were collected for subsequent tests. Protein concentration was determined using the bicinchoninic acid (BCA) assay (Sigma-Aldrich). Fifty microgram of proteins were separated by 10%–15% SDS-polyacrylamide gel followed by transferred onto 0.45-μm polyvinylidene difluoride membranes (Millipore, Billerica, MA, USA) at 100 mA for 1.5 h. The membrane was then blocked with 5% fat-free milk in TBS for 2 h at 37 °C, incubated with primary antibodies overnight at 4 °C and then with horseradish peroxidase (HRP)-conjugated secondary antibodies (goat anti-mouse and goat anti-rabbit; NeoBioscience, Shenzhen, China) for 1 h at 37 °C. Immunoreactive bands were treated with an ECL kit, visualized with an imaging system (Image station 4000M, Kodak, Rochester, NY, USA) and quantitatively analyzed using Image J software (Bethesda, MD, USA).

### 4.4. Primary Neuron Cultures

Primary olfactory bulb neurons were isolated from postnatal (P0-P1) pups of 3× Tg-AD mice. Briefly, olfactory bulbs were dissected from the brain and digested with 2 mg/mL papain for 30 min at 37 °C followed by dissociation with pipetting in DMEM with 10% FBS. Dissociated cells (5 × 10^5^ cells/per well) were plated in culture dishes coated with 100 µg/mL poly-d-lysine and cultured for 7 days in the neurobasal medium with 2% B27, 1× l-glutamine and 50 U/mL penicillin-streptomycin. At day 8, neurons were treated with 1 µM Se-Met for 24 h.

### 4.5. Statistical Analysis

Data were expressed as the mean ± SEM and all statistical analyses were performed by Student’s *t* test of the GraphPad Prism software (Lo Jolla, CA, USA). Statistical significance of the results was assessed at a level of *p* < 0.05.

## 5. Conclusions

In summary, we revealed that Se-Met could decrease the production and deposition of Aβ by inhibiting BACE1-regulated APP processing and reduce the level of total tau and phosphorylation of tau in the OB, which depends on the suppression of the activity and expression of GSK-3β and CDK5. Meanwhile, Se-Met could reduce glial activation, relieve the neuroinflammation and then attenuate neuronal cell death in the OB of 3× Tg-AD mice. To the best of our knowledge, this is the first evidence that Se-Met has a crucial protective effect on the OB in the early AD stage although further study concerning olfactory memory is required. We further demonstrated the therapeutic effect of Se-Met and verified the possibility that OB is also a feasible and sensitive tissue of pharmacological intervention in AD.

## Figures and Tables

**Table 1:** Antibody information.

Antibody	Host	Application	Source
APP	Mouse	IMB	Millipore (Billerica, MA, USA)
6E10	Mouse	IMB/IMF	Covance (Princeton, NJ, USA)
sAPPβ	Mouse	IMB/IMF	Covance
BACE1	Rabbit	IMB	Abcam (Cambridge, UK）
Tau5	Mouse	IMB/IMF	Abcam
Tau-pS404	Rabbit	IMB/IMF	Abcam
Tau-pS422	Rabbit	IMB	Abcam
GFAP	Rabbit	IMB/IMF	Abcam
CD45	Rabbit	IMB/IMF	Abcam
PSD95	Rabbit	IMB	Cell signaling (Danvers, MA, USA)
Synaptophysin	Rabbit	IMB	Cell signaling
Akt	Rabbit	IMB	Cell signaling
p-Akt	Rabbit	IMB	Cell signaling
CDK5	Rabbit	IMB	Cell signaling
GSK-3*β*	Mouse	IMB	Cell signaling
p-GSK-3*β*	Rabbit	IMB	Cell signaling
PP2A-C	Rabbit	IMB	Cell signaling
NeuN	Rabbit	IMF	Abcam
p-PP2A	Rabbit	IMB	Abcam
β-actin	Rabbit	IMB	Sigma (Santa clara, CA, USA)
α-tubulin	Mouse	IMB	Sigma
GAPDH	Rabbit	IMB	Proteintech (Rosemont, CA, USA)

APP, amyloid precursor protein; BACE1, CD45, cluster domain 45; GFAP, glial fibrillary acidic protein; GSK, glycogen synthase kinase; CDK5, Cyclin-dependent kinase 5; PSD95, postsynaptic density protein 95; PP2A, Protein Phosphatase 2A; IMB, Immunoblot; IMF, immunofluorescnce.

## Abbreviations

AD	Alzheimer’s disease
APP	amyloid precursor protein
CDK5	cyclin-dependent kinase 5
EP	The external plexiform layer
Gr	The granule cell layer
GSK-3*β*	glycogen synthase kinase-3*β*
IP	The inner plexiform layer
MT	The mitral and tufted cell layer
OB	Olfactory bulb
PHF	paired helical filaments
Se-Met	Selenomethionine
3× Tg-AD mice	Triple transgenic mouse model of AD
